# Neuromuscular electrical stimulation to augment lower limb exercise and mobility in individuals with spastic cerebral palsy: A scoping review

**DOI:** 10.3389/fphys.2022.951899

**Published:** 2022-08-30

**Authors:** Kelly R. Greve, Christopher F. Joseph, Blake E. Berry, Kornel Schadl, Jessica Rose

**Affiliations:** ^1^ Division of Occupational Therapy and Physical Therapy, Cincinnati Children’s Hospital Medical Center, Cincinnati, OH, United States; ^2^ Department of Rehabilitation, Exercise and Nutrition Sciences, University of Cincinnati, College of Allied Health Sciences, Cincinnati, OH, United States; ^3^ Department of Physical Therapy, Kennedy Krieger Institute, Baltimore, MD, United States; ^4^ Department of Orthopaedic Surgery, Stanford University, Stanford, CA, United States; ^5^ Motion and Gait Analysis Lab, Lucile Packard Children’s Hospital, Stanford Children’s Health, Stanford, CA, United States

**Keywords:** cerebral palsy, exercise, transcutanenous electric nerve stimulation, gait, neuromuscular electrical simulation

## Abstract

**Background:** Neuromuscular Electrical Stimulation (NMES) is an emerging assistive technology applied through surface or implanted electrodes to augment skeletal muscle contraction. NMES has the potential to improve function while reducing the neuromuscular impairments of spastic cerebral palsy (CP). This scoping review examines the application of NMES to augment lower extremity exercises for individuals with spastic CP and reports the effects of NMES on neuromuscular impairments and function in spastic CP, to provide a foundation of knowledge to guide research and development of more effective treatment.

**Methods:** A literature review of Scopus, Medline, Embase, and CINAHL databases were searched from 2001 to 2 November 2021 with identified inclusion and exclusion criteria.

**Results:** Out of 168 publications identified, 33 articles were included. Articles on three NMES applications were identified, including NMES-assisted strengthening, NMES-assisted gait, and NMES for spasticity reduction. NMES-assisted strengthening included the use of therapeutic exercises and cycling. NMES-assisted gait included the use of NMES to improve gait patterns. NMES-spasticity reduction included the use of transcutaneous electrical stimulation or NMES to decrease tone. Thirteen studies investigated NMES-assisted strengthening, eleven investigated therapeutic exercise and demonstrated significant improvements in muscle structure, strength, gross motor skills, walking speed, and functional mobility; three studies investigated NMES-assisted cycling and demonstrated improved gross motor skills and walking distance or speed. Eleven studies investigated NMES-assisted gait and demonstrated improved muscle structure, strength, selective motor control, gross motor skills, and gait mechanics. Seven studies investigated NMES for spasticity reduction, and five of the seven studies demonstrated reduced spasticity.

**Conclusion:** A growing body of evidence supports the use of NMES-assisted strengthening, NMES-assisted gait, and NMES for spasticity reduction to improve functional mobility for individuals with spastic CP. Evidence for NMES to augment exercise in individuals with spastic CP remains limited. NMES protocols and parameters require further clarity to translate knowledge to clinicians. Future research should be completed to provide richer evidence to transition to more robust clinical practice.

## 1 Introduction

Cerebral palsy (CP) is the most common motor disability in childhood, affecting 1.5 to 4 per 1,000 live births and presenting as spastic, dyskinetic, and ataxic types of CP, depending on the location of early brain injury (Bax et al., 2005). Spastic CP is the most common type of CP characterized by four interrelated neuromuscular impairments associated with corticospinal tract injury: muscle weakness, short muscle-tendon length relative to bone, spasticity, and impaired selective motor control (SMC) (Bax et al., 2005; Wright M. et al., 2012; Zhou et al., 2017). Dyskinetic CP is characterized by involuntary muscle contractions imposed on purposeful movement, limiting functional mobility, and is thought to be associated with basal ganglia injury (Sanger, 2015). Ataxic CP impairs balance and coordination associated with an injury in the cerebellum of the brain (Imamura et al., 1992; Rankin et al., 2010). Depending on the location of brain injury, an individual may present with symptoms of more than one type of CP (Schiariti et al., 2018). This review focuses on neuromuscular electrical stimulation (NMES) application to augment lower limb exercise for individuals with spastic CP, affecting around 80% of children with CP (Novak, 2014; CDC, 2020). Spastic CP can involve unilateral or bilateral limbs. In milder cases of CP, the lower limb is more affected distally, than proximally. Functional mobility in spastic CP is described by the Gross Motor Function Classification System (GMFCS). GMFCS levels range from I to V, with GMFCS I being mild and GMFCS V being the most severe (Palisano et al., 2007), and are reported in this review.

NMES is an emerging assistive technology applied as surface stimulation through electrodes placed over the skin or directly to the muscle via implanted electrodes to initiate or augment skeletal muscle contraction through intact peripheral nerves (Mooney and Rose, 2019; Wright et al., 2012). NMES applied through surface electrodes is the most common application as it is a non-invasive technique and generally well tolerated (Mooney and Rose, 2019). Electrodes are commonly placed over the motor point where the motor nerve innervates the muscle (Botter et al., 2011). The application of NMES to achieve functional movements is often referred to as Functional Electrical Stimulation (FES) (Masani and Popovic, 2011). The application of low-intensity electrical stimulation primarily targeting nerves, referred to as Transcutaneous Electrical Nerve Stimulation (TENS), is routinely used for pain management and has the potential to improve motor function in patients with neurodegenerative disorders (Levin and Hui-Chan, 1992; Vance et al., 2012; Kroeling et al., 2013). NMES applications include the use of NMES-assisted strengthening, NMES-assisted gait, and NMES spasticity reduction.

NMES parameters that control stimulation vary based on clinical application, targeted muscles, and individual tolerance (Maffiuletti, 2010). Parameters reported in this review include stimulation frequency, intensity, pulse width, timing (on/off ratio), and ramp. The frequency of electrical stimulation refers to the number of times a pulse of current is applied within one second, measured in Hertz (Hz). Higher frequencies generally produce more muscle activation as long as the individual pulses reach muscle fibers after their refractory period, do not result in neurotransmitter depletion, or do not block nerves otherwise (e.g., nerve blocking with monophasic high-frequency stimulation or with charge-balanced kilohertz frequency alternating current), therefore, it generates more force and can lead to increased fatigue and lower tolerance (Chaudhuri and Behan, 2004; Gorgey et al., 2009; Wegrzyk et al., 2015). Intensity or pulse amplitude refers to the amount of current delivered, or the voltage applied to the electrodes (respectively resulting in change of the current delivered) during each pulse. It is measured in milliamperes (mA) for current-controlled and Volts for voltage-controlled stimulation, where the current is proportional to the voltage. Pulse width refers to the duration between the start and end of each electrical pulse and is typically reported in microseconds (μs). Longer pulse widths are associated with increased muscle force; however, shorter pulse widths may provide patients with more comfort and increased tolerance (Mogyoros et al., 1996; Knash et al., 2003; Mang et al., 2011, 2011). Timing (on/off) refers to the duration the stimulation with a given frequency is turned on versus turned off, typically reported in seconds, whereas ramp refers to the gradual increase followed by a gradual decrease in stimulation intensity to facilitate adaptation, reduce the likelihood of discomfort, and promote smooth gradations of tetany between different muscle groups (Baker et al., 2000; Bijak et al., 2005).

A growing body of evidence supports the use of NMES in the treatment and care of individuals with CP (Mooney and Rose, 2019; Novak et al., 2020). In this review, treatments were categorized into NMES-assisted strengthening exercises (therapeutic exercise and cycling), NMES-assisted gait (overground and treadmill walking for neuroprosthetic and neurotherapeutic effects), and NMES for spasticity reduction (during strengthening exercise and gait which typically targets spastic muscles with lower frequency stimulation using TENS parameters). The ultimate goal of NMES for individuals with CP is to improve functional mobility and quality of life.

Muscle weakness is a common impairment in individuals with CP and significantly impacts their ability to function and participate in activities. Weakness is primarily caused by neurological impairment, including reduced motor-unit firing and by muscle structural changes including in the muscle fascicles such as fatty replacement, in sarcomeres, and in muscle fiber size variability (Huijing, 1998; Elder et al., 2003; Lieber et al., 2004; Foran et al., 2005; Rose and McGill, 2005; Malaiya et al., 2007; Stackhouse et al., 2007; Barber et al., 2012; Noble et al., 2014; Zhou et al., 2017). Evidence indicates that use of NMES for augmenting exercise increases microvascular perfusion in the stimulated skeletal muscle (Clemente et al., 1991; Moloney et al., 2006; Bahadori et al., 2017). This decreases the diffusion distance in the stimulated muscle tissue and enhances the exchange of nutrients and metabolites between the blood and tissue, improving physiological muscle function. Given the vital role of muscle tissue (e.g., in maintaining stable glucose metabolism), NMES might further benefit the overall quality of life in individuals across all GMFCS levels.

Accurate interpretation of research requires relevant, validated outcome measures. Therefore, this review includes studies that report outcome measures recommended as Common Data Elements (CDE) by The National Institute of Neurological Disorders and Stroke (NINDS) (Grinnon et al., 2012). The CDE database is structured by diagnosis and includes CDEs recommended for CP.

Using the NINDS CDE database, there are several ways to measure and assess changes in strength in individuals with CP (Table 1). These include both direct strength measures, such as Manual Muscle Testing and Maximum Voluntary Isometric Contraction Testing, as well as measures of functional mobility, such as temporal-spatial parameters of gait (Lee et al., 2008), 3D gait analysis of kinematics and kinetics including the Gait Deviation Index (GDI) (Schwartz and Rozumalski, 2008), 6 Minute Walk Test (6MWT) (Maher et al., 2008) which reflects gait distance, Timed Up and Go (TUG) (Kaya Kara et al., 2019), and Gross Motor Function Measure (GMFM) (Russell et al., 2000). Although not CDE outcomes, dynamometry and timed sit to stand are often used to reflect changes in muscle strength and function in individuals with CP. Changes in muscle physiology can be assessed indirectly through muscle structure using musculoskeletal ultrasound (US) and Magnetic Resonance Imaging (MRI). Our review also identified in certain studies the CDE measures of Selective Control Assessment of the Lower Extremity (SCALE) (Fowler et al., 2009) for assessment of SMC.

**TABLE 1:** NINDS Common Data Elements (Grinnon et al., 2012) outcome measures identified in the articles reviewed, assessing motor function, spasticity, movement, functional mobility, and Quality of Life.

US	Ultrasound
MRI	Magnetic Resonance Imaging
6MWT (Maher et al., 2008)	6 Minute Walk Test
TUG (Kaya Kara et al., 2019)	Timed Up and Go
WS	Walking Speed
IGA	Instrumented Gait Analysis
GDI (Schwartz and Rozumalski, 2008)	Gait Deviation Index
SAGV	Stride Analysis and Gait Variability
OGS (Mackey et al., 2003)	Observational Gait Scale
GMFM (Russell et al., 2000)	Gross Motor Function Measure
PEDI (Haley, 1992)	Pediatric Evaluation of Disability Inventory
LAQ (Mackie et al., 1998)	Lifestyle Assessment Questionnaire
PEM-CY (Coster et al., 2010)	Participation and Environment Measure for Children and Youth
WeeFIM (Ottenbacher et al., 2000)	Functional Independent Measure for Children
COPM (Law et al., 1990)	Canadian Occupational Performance Measure
SCALE (Fowler et al., 2009)	Selective Control Assessment of the Lower Extremity
MAS (Mutlu et al., 2008)	Modified Ashworth Scale
TS (Gracies et al., 2010)	Tardieu Scale

Common data elements (CDE) by the national institute of neurological disorders and stroke (NINDS).

This scoping review examines the application of NMES to augment lower extremity exercises for individuals with spastic CP, and reports the effects of NMES on neuromuscular impairments and function in spastic CP, to provide a foundation of knowledge that can guide research to advance the field and provide more effective treatment.

## 2 Methods

Given the extent of the literature, we determined that the most appropriate type of review for this field is a scoping review (Pollock et al., 2022). The primary goal of our review was to give a comprehensive assessment of the current use of NMES for augmenting exercise for individuals with spastic CP. We also sought to identify knowledge gaps to guide future research directions. The Preferred Reporting Items for Systematic Reviews and Meta-Analyses extension for Scoping Reviews (PRISMA-ScR) checklist was utilized to guide this review (Tricco et al., 2018).

A literature search was completed using Scopus, Medline, Embase, and CINAHL databases with additional publications referenced through the primary search. The search was completed on 2 November 2021, using the following keywords and Boolean operators: “spastic cerebral palsy” AND “neuromuscular electrical stimulation” OR “functional electrical stimulation”. The inclusion criteria for the articles were as follows: 1) the study involved individuals with CP, 2) the study reported outcome measures recommended by CDE for CP and were related to muscle strength and function, gait temporal-spatial parameters, and kinematics as identified in Table 1; 3) the study incorporated a known NMES dosage (session, duration, and frequency) with a known exercise component, such as strengthening, cycling, gait training; 4) the study was available in English; and 5) the study was published as a full-text manuscript. The exclusion criteria for the articles were as follows: 1) NMES was not a component of the study, 2) exercise was not a component of the study, 3) duration of treatment period was less than 4 weeks or not reported; 4) investigated muscles were not involving lower extremities; 5) articles were from dissertations, conference posters, or abstracts, 6) studies were published before 2001.

Using recommendations by the National Institute of Neurological Disorders and Stroke (NINDS), the authors used publications reporting at least one common data element (CDE) outcome measures specific to the diagnosis of CP. Each publication was given a level of evidence based on the Oxford Centre for Evidence-Based Medicine 2011 Level of Evidence guidelines (Howick, 2011). Data were extracted by the authors (KG, CJ, KS, BB) for each publication but unblinded to the results of other authors.

## 3 Results

**FIGURE 1:**
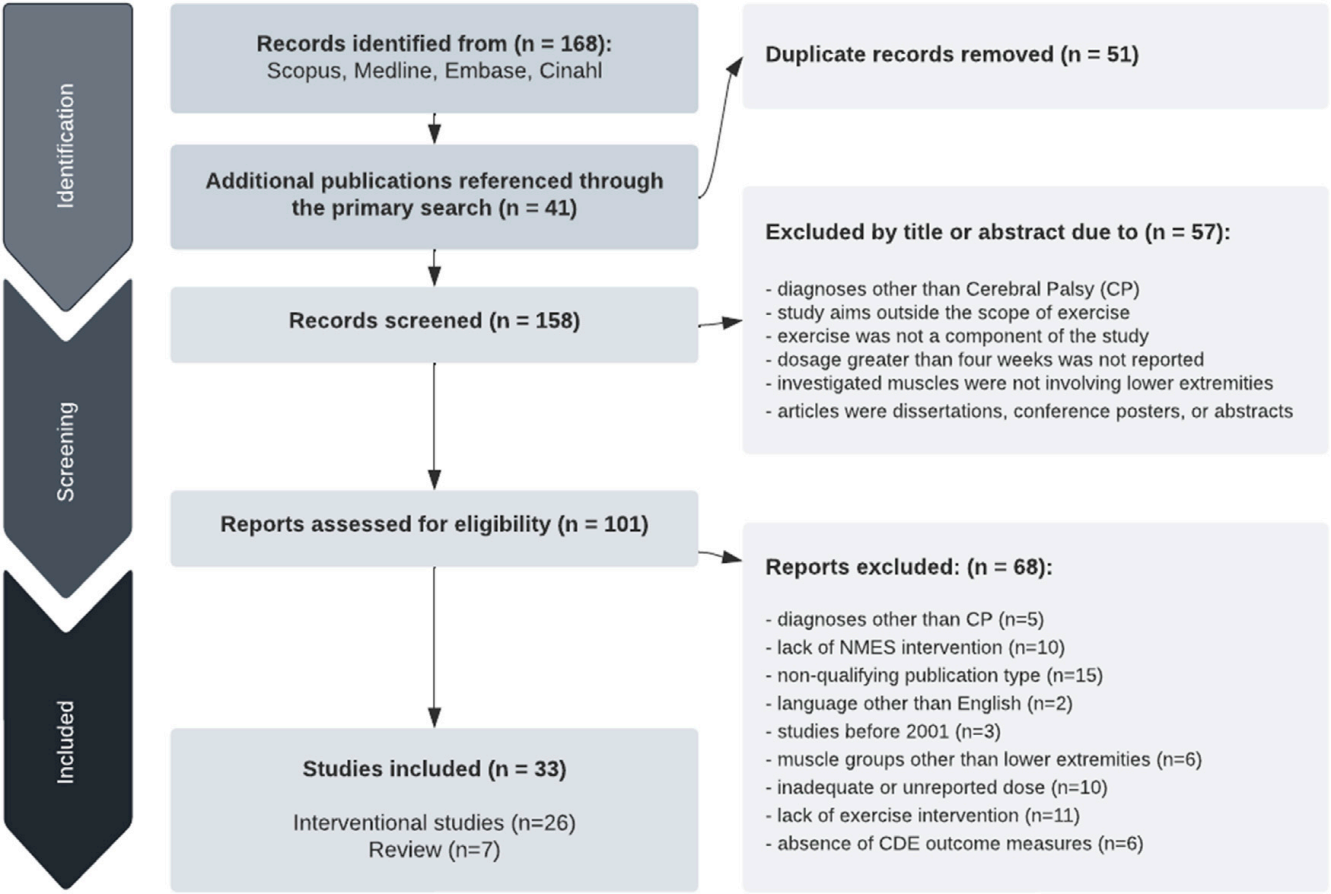
PRISMA flowchart of the study selection process.

The initial 5-database search resulted in 168 publications, and an additional 41 articles were identified from references. Fifty-one articles were duplicates. The authors used titles and abstracts to screen the publications for the relevance of exercise programs involving the lower extremity. Fifty-seven articles were discarded due to diagnoses other than spastic CP or study aims outside the scope of exercise. One hundred and one articles, including seven review articles, met criteria and were fully reviewed by the authors; however, 68 were excluded upon further review for different populations (*n* = 5), absence of CDE for CP outcome measures (*n* = 6), lack of NMES intervention (*n* = 10), inadequate or unreported treatment duration (*n* = 10), lack of exercise component (*n* = 11), language other than English (*n* = 2), muscle groups other than lower extremities (*n* = 6), non-qualifying publication type (n = 15), and published before 2001 (*n* = 3). Based on these inclusion criteria, this scoping review includes a total of 33 articles, 26 intervention studies, and seven reviews. See Figure 1 for the publication search flow chart.

The literature was categorized by the application of NMES, including NMES-assisted strengthening, NMES-assisted gait, and NMES for spasticity reduction. Extracted variables included the study’s aim study design, age of participants, sample size, limb involvement (bilateral and/or unilateral), GMFCS level, device type, targeted muscle, NMES dose (number of weeks, sessions per week, and time per session), NMES parameters (frequency, intensity, pulse width, timing, ramp, waveform, and mode), and CDE outcomes recommended by NINDS, detailed in Tables 2–4.

**TABLE 2:** Articles reviewed reporting level of evidence, participant characteristics, NMES intervention, and outcomes measures.

Intervention/Authors (year)	NMES Intervention	Evidence Level	Study Design	Age (years)	Sample Size	GMFCS Level	Limbs	Muscle	NMES Duration (weeks)	Frequency of use (days/week)	Session duration (min)
**Strengthening**											
Arya et al. (2012)	Strengthening	2	RCT	7–14	10	-	Bilateral, Unilateral	Quads, TA	4	4–5	20–30
Daichman et al. (2003)	Strengthening, Spasticity Reduction	4	CR	13	1	-	Bilateral	Quads	6	3–4	5–15
Greve and Colvin, 2021)	Strengthening	4	CS	9–15	3	II	Bilateral	Quads	6	7	15–30
Karabay et al. (2015)	Strengthening, Spasticity Reduction	3	PCS	3–14	28	I-V	Bilateral	GS, TA	4	5	30
Kerr et al. (2006)	Strengthening	2	RCT	5–16	60	-	Bilateral	Quads	16	5	60
Khalili and Hajihassanie, 2008	Strengthening, Spasticity Reduction	2	RCT	11–14	11	-	Bilateral	Quads	4	3	30
Mukhopadhyay et al. (2017)	Strengthening	3	PCS	7–14	26	I-III	Bilateral, Unilateral	TA	12	5	30
Nunes et al. (2008) (Nunes et al., 2008)	Strengthening	3	PT	7–15	10	-	Unilateral	TA	7	1–2	30
Qi et al. (2018)	Strengthening	2	RCT	4–9	100	-	-	TA	6	5	20
Rajalaxmi et al. (2017)	Strengthening, Spasticity Reduction	3	PT	5–10	30	-	Bilateral	TA	8	5	15–20
Stackhouse et al. (2007)	Strengthening	3	PS	8–12	11	II-III	Bilateral	Quads, GS	12	3	15/muscle
Armstrong et al. (2020)	Cycling	2	RCT	6–18	21	II-IV	Bilateral, Unilateral	Gluteals, Quads, HS, GS, TA	8	3	30
Johnston and Wainwright, 2011	Cycling	4	CR	49	1	II	Bilateral	Gluteals, Quads, HS, GS	12	3	30
Özen et al. (2021)	Cycling, Spasticity Reduction	2	RCT	4–12	25	I-III	Bilateral	Quads, HS, GS, TA	4	5	30
**Gait**											
Chan et al. (2004)	Gait	4	SSRD	4–11	12	-	Bilateral, Unilateral	GS	4	3	15
Damiano et al. (2013)	Gait	3	PT	8–19	14	I-II	Bilateral, Unilateral	TA	40	7	360
Gonçalves et al. (2019)	Gait	4	SSRD	4–7	4	I-II	Unilateral	GS	8	3	50
Johnston et al. (2004)	Gait	3	PT	6–12	17	II-IV	Bilateral	Hip Add., Gluteals, Quads, HS, GS, TA	4	5	≤60
Pool et al. (2014) (Pool et al., 2014)	Gait	4	SSRD	5–18	12	I-II	Unilateral	TA	8	6	≥60
Pool et al. (2015)	Gait, Spasticity Reduction	2	RCT	5–18	32	I-II	Unilateral	TA	8	6	≥240
Pool et al. (2016)	Gait	2	RCT	5–18	32	I-II	Unilateral	TA	8	6	≥240
Prosser et al. (2012)	Gait	3	PT	7–19	19	I-II	Unilateral	TA	12	6	30–360
Robinson et al. (2015)	Gait	4	CR	57	1	-	Bilateral	HS, TA	6	5	480
van der Linden et al. (2003)	Gait	2	RCT	5–14	22	-	Bilateral, Unilateral	Gluteals	8	6	30–60
van der Linden et al. (2008)	Gait	2	RCT	4–15	14	-	Bilateral, Unilateral	Quads, TA	10	6	60
**Spasticity Reduction**											
AlAbdulwahab and Al-Gabbani, 2010	Spasticity Reduction	3	RCT	7–12	42	-	Bilateral	Hip Add	1	7	3 × 15
Daichman et al. (2003)	Strengthening, Spasticity Reduction	4	CR	13	1	-	Bilateral	Quads	6	3–4	5–15
Karabay et al. (2015)	Strengthening, Spasticity Reduction	3	PCS	3–14	28	I-V	Bilateral	GS, TA	4	5	30
Khalili and Hajihassanie, (2008)	Strengthening, Spasticity Reduction	2	RCT	11–14	11	-	Bilateral	Quads	4	3	30
Özen et al. (2021) (Özen et al., 2021)	Cycling, Spasticity Reduction	2	RCT	4–12	25	I-III	Bilateral	Quads, HS, GS, TA	4	5	30
Pool et al. (2015)	Gait, Spasticity Reduction	2	RCT	5–18	32	I-II	Unilateral	TA	8	6	≥240
Rajalaxmi et al. (2017)	Strengthening, Spasticity Reduction	3	PT	5–10	30	-	Bilateral	TA	8	5	15–20

Level of Evidence (Howick, 2011).

Study design abbreviations: Case Report (CR) Case Series (CS), Pilot Study (PS), Prospective Trial (PT), Prospective Controlled Study (PCS), Randomized Controlled Trial (RCT), Single Subject Research Design (SSRD).

Muscle abbreviations: Gluteus Maximus and/or Medius (Gluteals), Quadriceps (Quads), Tibialis Anterior (TA), Gastrocnemius & Soleus (GS), Hamstrings (HS).

CDE, outcome measures abbreviations: Refer to Table 1.

Other outcome measure abbreviations: Physiological Cost Index (PCI), Selective Motor Control (SMC), Australian Spasticity Assessment Scale (ASAS), Activities-specific Balance Confidence scale (ABC), Tinetti Performance Oriented Mobility Assessment (POMA).

**TABLE 3:** Articles reviewed reporting NMES parameters.

Authors (year)	NMES frequency (Hz)	NMES intensity (mA)	NMES pulse width (μs)	NMES Timing [on/off] (sec)	NMES ramp [up/Down] (sec)	NMES Waveform	NMES mode
AlAbdulwahab and Al-Gabbani, (2010)	100	Until tingling sensation	250	-	-		Constant
Armstrong et al. (2020)	40–50	Tolerance	200–250	-	-	-	-
Arya et al. (2012)	20–40	Tolerance	200	14/5	3	Biphasic	Alternate
Chan et al. (2004)	30–35	Visible muscle contraction	-	-	-	-	Manually triggered during stance
Daichman et al. (2003)	35	Tetanic contraction	300	10/50	2	-	-
Damiano et al. (2013)	25	-	25–50	-	-	Asymmetric, Biphasic	Timed with gait
Gonçalves et al. (2019)	26–30	17–33	300	-	-	Symmetric	Manually triggered during activities
Greve and Colvin, (2021)	35	9.75–32.5	200–350	5–10/10–30	1–2	Symmetric, Biphasic	Synchronous
Johnston et al. (2004)	20	20	200	2–4/0	1–3	Asymmetric, Biphasic	-
Johnston and Wainwright, (2011)	33	40–80	250	-	-	-	-
Karabay et al. (2015)	25	20–30	250	10/12	-	-	-
Kerr et al. (2006) (Kerr et al., 2006)	35	Tolerance	300	7/12	2/1	-	-
Khalili and Hajihassanie, (2008)	30	Visible muscle contraction	400	4/4	0.5	-	-
Mukhopadhyay et al. (2017)	40	0–30	200	-	-	Biphasic	-
Nunes et al. (2008)	50	28–44	250	5/10	-	-	-
Özen et al. (2021)	30–45	100	250–300	-	7/2	Biphasic	-
Pool et al. (2014)	33	Tolerance	300	-	-	Asymmetric, Biphasic	-
Pool et al. (2015)	33	-	25–100	-	-	Asymmetric, Biphasic	-
Pool et al. (2016)	33	-	25–100	-	-	Asymmetric, Biphasic	-
Prosser et al. (2012)	16.7–33	-	25–300	-	-	Asymmetric, Biphasic	Timed with gait
Qi et al. (2018)	-	Visible muscle contraction	-	-	-	-	Constant
Rajalaxmi et al. (2017)	-	-	-	-	-		-
Robinson et al. (2015)	30–40	Tolerance	200–300	-	-	Symmetric, Biphasic	Timed with gait
Stackhouse et al. (2007)	50	20	5–200	15/45	3		Alternate
van der Linden et al. (2003)	10–30	-	75–100	5/10–15	0.8	Asymmetric, Biphasic	-
van der Linden et al. (2008)	10–40	20–70	3–350	6/10–14	0.8	Asymmetric, Biphasic	Triggered during gait

**TABLE 4:** Articles reviewed reporting NINDS common data elements (CDE) and other outcome measures.

Authors (year)	CDE Outcome measures	Change in CDE outcome measures relative to control	Other Outcome measures	Change in other outcome measures relative to control
AlAbdulwahab and Al-Gabbani, (2010)	WS	WS ↑ (*p* < 0.021), Step length ↑ (*p* < 0.008)	Visual observations of knee positions	Visual observations of knee positions (improved) ↑
	SAGV	SAGV ↑ (improved)		
	MAS	MAS ↓ (hip adduction spasticity decreased *p* < 0.001)		
Armstrong et al. (2020)	GMFM	GMFM ↑ (*p* < 0.001)	Sit to Stand	
	PEDI-CAT	PEDI-CAT (no change)		
	PEM-CY	PEM-CY (no change)		
	COPM	COPM ↑ (*p* < 0.001)		
Arya et al. (2012)	WS	WS: 7.83 m/min (*p* < 0.01) ↑	Physiological Cost Index (PCI)	PCI: 1.83 (*p* < 0.001) ↓
	SAGV	Cadence: 23.33 steps/m (*p* < 0.01) ↑		EMG (no change)
	GMFM	GMFM (no significant difference)		
Chan et al. (2004)	IGA	GMFM ↑ (*p* < 0.003)	-	-
	GMFM	IGA ↑ (Improved ankle power *p* = 0.015)		
Daichman et al. (2003)	SAGV	SAGV (walking velocity, step length, and cadence) ↑	Range of motion (ROM)	ROM ↑ (popliteal angle decreased from 40 to 35°)
	PEDI	PEDI ↑	Dynamometry	Dynamometry (quads strength ↑ from 16.3 N to 33.7 N)
	MAS	MAS (no significant difference)		
Damiano et al. (2013)	US	TA (US) CSA ↑	-	-
	IGA	IGA (no change)		
Gonçalves et al. (2019) (Gonçalves et al., 2019)	WS	WS ↑, GMFM ↑	-	-
	GMFM			
Greve and Colvin, (2021)	6MWT	6MWT ↑ (above MCID)	-	-
	GMFM	GMFM ↑ (above MCID)		
	FMS	FMS ↑		
Johnston et al. (2004)	SAGV	SAGV (Walking velocity, step length, and cadence ↑ [*p* < 0.05])	ROM	ROM ↑ (*p* < 0.05)
	GMFM	GMFM ↑ (*p* < 0.05)	VO2/kg/m	VO2/kg/m (no change)
Johnston and Wainwright, (2011)	6MWT	6MWT (didn’t meet MDC)	ROM	Dynamometry (22% quads and 18.5% HS strength ↑)
	TUG	TUG ↓ (from 11.9 to 9.0 s)	Dynamometry	
	SAGV	SAGV	Bioimpedance monitor	
	COPM	COPM ↑ - barefoot gait speed of 0.09 m/s and in step length of 0.03–0.05 m (likely not clinically meaningful)	McGill-Melzack Pain Questionnaire	
Karabay et al. (2015) (	US	US (CSA) ↑ (TA from 238.7 to 282.0 mm^2^, *p* < 0.001; GS from 207.9 to 229.5 mm^2^, *p* < 0.008)	ROM	ROM (no change)
	MAS	MAS (no change)		
Kerr et al. (2006)	GMFM	GMFM (no change)	Dynamometer	Dynamometer (no change)
	LAQ	LAS (from LAQ-CP) ↓ (placebo: 39.98, TES: 33.98, *p* < 0.05)		
Khalili and Hajihassanie, (2008)	MAS	MAS ↓ (2.0 compared to 1.2 in the control group, *p* = 0.046)	ROM	ROM ↑ (from 9 to 13°, *p* = 0.04)
Mukhopadhyay et al. (2017)	WS	WS ↑ (17.67%)	PCI	PCI ↓ (19.7%)
	SAGV	SAGV ↑ step length ↑ (4.08%)		
	GMFM	cadence ↑ (16.17%)		
		GMFM ↑ (2.1%)		
Nunes et al. (2008)	GMFM	GMFM ↑ (group 1: from 94.28% to 97.14% *p* < 0.05, group 2: from 95.23% to 98.09% *p* < 0.05)	ROM	ROM ↑ (group 1: active and passive ankle dorsiflexion *p* = 0.05, group 2: passive ankle dorsiflexion *p* < 0.05)
				TA muscle strength of ↑ (manual)
Özen et al. (2021)	6MWT	6MWT ↑	Visual Gait Analysis	Visual Gait Analysis ↑ (improvement in ankle dorsiflexion and foot contact)
	GMFM	GMFM ↑		
	WeeFIM	WeeFIM ↑		
	MAS	MAS ↓		
	Tardieu Scale	Tardieu Scale ↓		
Pool et al. (2014)	OGS	OGS (no change)	ROM	ROM ↑ (*p* < 0.01)
			Dynamometry	Dynamometry ↑ (*p* < 0.01)
			Australian Spasticity Assessment Scale (ASAS)	ASAS ↓ (*p* < 0.01)
			SMC dorsiflexion grade (Boyd and Graham, 1999)	SMC dorsiflexion grade ↑
				Self-reported Toe Drag ↓ (*p* = 0.02) and Falls ↓ (*p* < 0.01)
Pool et al. (2015)	IGA	IGA ↑:	ASAS	ASAS ↓ (p = 0.038)
	Tardieu Scale	- ankle angle ↑ (mean difference 11.9°, 95% CI 6.8°–17.1°, *p* < 0.001)	Community Balance and Mobility Scale	Community Balance and Mobility Scale ↑ (mean difference 8.3,
		- stance ↑ (mean difference 0.27, 95% CI 0.05–0.49, *p* = 0.011)	4-Square Step Test	95% CI 3.2–13.4; *p* < 0.001),
		- step length ↑ (mean difference 0.06, 95% CI 0.003–0.126, *p* = 0.035)		4-Square Step Test (no significant change, *p* = 0.182),
		Tardieu Scale ↑ (dynamic ankle dorsiflexion range mean difference 6.9°, 95% CI 0.4°–13.6°, *p* = 0.035)		Self-report Toe Drag (*p* = 0.002) and Falls ↓ (toe dragging: *p* = 0.002, falling: *p* = 0.022)
Pool et al. (2016)	MRI	MRI ↑ (TA muscle volume, *p* = 0.039)	Dynamometry	Dynamometry:
	SCALE	SCALE ↑ (mean difference 0.81, 95% CI 0.3–1.32, *p* < 0.001)		- TA strength ↑ (*p* = 0.002)
				- Ankle SMC ↑ (median difference 0.5, IQR 0–1, *p* = 0.048)
Prosser et al. (2012)	IGA	IGA ↑ (mean and peak dorsiflexion during swing and at foot-floor contact)	-	-
		WS (no change)		
Qi et al. (2018)	WS	WS ↑ (0.72 m/s vs. 0.57 m/s, *p* < 0.05)	Comprehensive Spasticity Scale score	Comprehensive Spasticity Scale score ↓ (7.4 vs. 9.4, *p* < 0.05)
	GMFM	GMFM ↑ (71 vs. 58, *p* < 0.05)		
Rajalaxmi et al. (2017)	MAS	MAS ↓ (*p* < 0.001)	ROM	ROM ↑ (AROM of dorsiflexors, *p* < 0.001; PROM, *p* < 0.001)
			Cadence	Cadence ↑
Robinson et al. (2015)	OGS	OGS ↑ (from 12/22 to 19/22 [right], 14/22 to 21/22 [left])	Activity-specific Balance Confidence (ABC) Scale	ABC ↑ (from 32.8% to 48.1%),
			Performance Oriented Mobility Assessment (POMA)	POMA ↑ (from 12/28 to 15/28),
			Dynamic Gait Index	Dynamic Gait Index ↑ (from 6/24 to 14/24)
Stackhouse et al. (2007)	MRI	MRI ↑ (CSA of Quads +4.42 cm^2^, *p* = 0.023)	Dynamometry	Dynamometry ↑ (MVIC ↑ from 81.8% to 118.9%, voluntary muscle activation of Quads ↑, +0.057, *p* = 0.084)
	WS	WS ↑ (*p* = 0.028)		
	IGA	IGA		
van der Linden et al. (2003)	IGA	IGA (no change)	Myometer	Myometer ↑ (strength, not significant)
	GMFM	GMFM ↑ (not significant)	ROM	ROM (no change)
			Parent Questionnaire	Parent Questionnaire (64% of the parents thought that the treatment made a difference to their child)
van der Linden et al. (2008)	IGA	IGA ↑ (*p* < 0.01)	Functional Assessment Questionnaire	Functional Assessment Questionnaire
		WS ↓ (0.03 m/s, *p* < 0.05)		

Common data elements (CDE) by the national institute of neurological disorders and stroke (NINDS).

### 3.1 Neuromuscular electrical stimulation-assisted strengthening

A total of fourteen articles were included for NMES-assisted strengthening, as shown in Table 2. NMES-assisted strengthening interventions included NMES augmenting therapeutic exercise, pre-operative surgical preparation, post-operative recovery, and NMES-assisted cycling. Several articles overlapped in the type of intervention, such as strengthening and spasticity reduction.

#### 3.1.1 Neuromuscular electrical stimulation-assisted therapeutic exercise

Eleven studies reported NMES-assisted therapeutic exercise intervention: one case report (Daichman et al., 2003), one case series (Greve and Colvin, 2021), one pilot study (Stackhouse et al., 2007), two prospective trials (Nunes et al., 2008; Rajalaxmi et al., 2017), two prospective controlled studies (Karabay et al., 2015; Mukhopadhyay et al., 2017), and four randomized controlled trials (RCT) (Kerr et al., 2006; Khalili and Hajihassanie, 2008; Arya et al., 2012; Qi et al., 2018). Strengthening involved both home and clinic interventions using portable NMES devices with surface or implanted electrodes focused on the quadriceps, gastrocnemius, and tibialis anterior muscles. NMES was applied during positioning, stretching, facilitated exercises, strengthening, activities of daily living, balance, posture, and gait exercises (Daichman et al., 2003; Kerr et al., 2006; Stackhouse et al., 2007; Khalili and Hajihassanie, 2008; Nunes et al., 2008; Arya et al., 2012; Karabay et al., 2015; Mukhopadhyay et al., 2017; Rajalaxmi et al., 2017; Qi et al., 2018; Greve and Colvin, 2021). Dosage consisted of 15–60 min, one to seven times per week for 4–16 weeks. See Table 3 for specific NMES parameters and dosage for each study.

Ten studies using NMES-assisted therapeutic exercise reported improvements in muscle structure, strength, gross motor skills, WS, and functional mobility (Daichman et al., 2003; Stackhouse et al., 2007; Khalili and Hajihassanie, 2008; Nunes et al., 2008; Arya et al., 2012; Karabay et al., 2015; Mukhopadhyay et al., 2017; Rajalaxmi et al., 2017; Qi et al., 2018; Greve and Colvin, 2021). Two studies examined muscle cross-sectional area (CSA) using ultrasound or MRI and found an increase in CSA values of the quadriceps (Stackhouse et al., 2007), tibialis anterior (Karabay et al., 2015), and gastrocnemius (Karabay et al., 2015). Two studies reported an increase in quadriceps strength assessed with dynamometry (Daichman et al., 2003; Stackhouse et al., 2007). Six studies conducted the GMFM (Kerr et al., 2006; Nunes et al., 2008; Arya et al., 2012; Mukhopadhyay et al., 2017; Qi et al., 2018; Greve and Colvin, 2021), and four of the six studies reported positive changes in gross motor skills (Nunes et al., 2008; Mukhopadhyay et al., 2017; Qi et al., 2018; Greve and Colvin, 2021). Two studies reported improvement in functional mobility using the PEDI (Daichman et al., 2003) and FMS (Greve and Colvin, 2021). Studies also reported improvement in gait (Daichman et al., 2003; Arya et al., 2012; Mukhopadhyay et al., 2017; Rajalaxmi et al., 2017), WS (Stackhouse et al., 2007; Arya et al., 2012; Mukhopadhyay et al., 2017; Qi et al., 2018), and endurance (Greve and Colvin, 2021) following NMES. Five studies (Daichman et al., 2003; Kerr et al., 2006; Stackhouse et al., 2007; Khalili and Hajihassanie, 2008; Greve and Colvin, 2021) commented on adherence with 90–100% tolerance for using NMES by individuals participating in these studies. See Table 4 for CDE outcomes and results of each study.

#### 3.1.2 Neuromuscular electrical stimulation-assisted cycling

Three studies reported NMES-assisted cycling for exercise, where multichannel NMES was applied using surface electrodes while the participant rode an indoor tricycle or stationary bicycle. One case report (Johnston and Wainwright, 2011) and two RCTs (Armstrong et al., 2020; Özen et al., 2021) reported on multichannel NMES used to target multiple muscles during cycling, including the gluteals, quadriceps, hamstrings, gastrocnemius, and/or anterior tibialis. NMES was applied during cycling alone or in addition to interventions, such as ROM, strengthening, and balance. NMES intervention dosage ranged from 30 min, 3–5 times per week for 4–12 weeks.

Three studies using NMES-assisted cycling reported boosting gross motor skills, walking distance, and speed (Johnston and Wainwright, 2011; Armstrong et al., 2020; Özen et al., 2021). Two studies (Armstrong et al., 2020; Özen et al., 2021) reported improvement in gross motor skills assessed with the GMFM. Studies also reported an increase in walking distance assessed with 6MWT (Johnston and Wainwright, 2011; Özen et al., 2021) and speed assessed using the TUG (Johnston and Wainwright, 2011). NMES was well-tolerated in one study (Özen et al., 2021) and variable in two studies (Johnston and Wainwright, 2011; Armstrong et al., 2020). See Tables 2, 3, 4 for details of each study’s NMES application and CDE outcomes.

### 3.2 Neuromuscular electrical stimulation-assisted gait

Table 2 reports the results of NMES-assisted gait, which includes interventions using NMES during gait for treadmill or overground walking with a known therapeutic dosage.

Eleven studies reported NMES-assisted gait for strengthening and improving gait pattern, including one case report (Robinson et al., 2015), three single-subject research design studies (SSRD) (Chan et al., 2004; Pool et al., 2014; Gonçalves et al., 2019), three prospective trials (Johnston et al., 2004; Prosser et al., 2012; Damiano et al., 2013), and four RCTs (Pool et al., 2016; 2015, van der Linden et al., 2003, 2008). Various NMES devices were used, including surface electrodes for non-wearable units targeting the gluteals, quadriceps, gastrocnemius, and tibialis anterior. Wearable units targeted hip adductors, gluteus maximus and medius, quadriceps, tibialis anterior, and gastrocnemius. NMES was applied during walking overground or performing functional task training. Only one study applied NMES while on a treadmill (Chan et al., 2004). NMES dosage ranged from 15 min to 8 h per day, 3–7 days per week for 4–40 weeks. See Table 3 for details of each study’s NMES application and parameters.

The eleven studies that investigated NMES-assisted gait found improved muscle structure, strength, SMC, gross motor skills, and gait (van der Linden et al., 2003, 2008; Chan et al., 2004; Johnston et al., 2004; Prosser et al., 2012; Damiano et al., 2013; Pool et al., 2016, 2015, 2014; Robinson et al., 2015; Gonçalves et al., 2019). NMES-assisted gait resulted in increased muscle volume of tibialis anterior as assessed on MRIs (Pool et al., 2016), increased tibialis anterior CSA as assessed on ultrasound (Damiano et al., 2013), increased strength as assessed by dynamometers (Pool et al., 2016, 2014), improved SMC as assessed by SCALE (Pool et al., 2016), improved gross motor skills as assessed by GMFM (van der Linden et al., 2003; Chan et al., 2004; Johnston et al., 2004; Gonçalves et al., 2019), and improved gait as assessed by kinematics, kinetics, and temporal-spatial parameters (Chan et al., 2004; Johnston et al., 2004; van der Linden et al., 2008; Prosser et al., 2012; Pool et al., 2015; Robinson et al., 2015). Compliance was reported to be high for NMES intervention (Chan et al., 2004; Prosser et al., 2012; Pool et al., 2016, 2015). Tolerance was reported as ranging from good (Damiano et al., 2013; Pool et al., 2014) to variable (van der Linden et al., 2003, 2008). See Table 4 for CDE outcome measures and results for each NMES-assisted gait study.

### 3.3 Neuromuscular electrical stimulation for spasticity reduction

Seven studies reported on the effects of NMES on spasticity. One case report (Daichman et al., 2003), one prospective controlled study (Karabay et al., 2015), one prospective trial (Rajalaxmi et al., 2017), and four RCTs (Khalili and Hajihassanie, 2008; AlAbdulwahab and Al-Gabbani, 2010; Pool et al., 2015; Özen et al., 2021). The targeted muscles for NMES included hip adductors, quadriceps, hamstrings, gastrocnemius, and tibialis anterior. NMES was applied to the antagonist muscle during exercises, including ROM, balance, strengthening, and gait training (Daichman et al., 2003; Khalili and Hajihassanie, 2008; Pool et al., 2015; Rajalaxmi et al., 2017; Özen et al., 2021). TENS was applied to the antagonist muscle during ROM and gait training exercises (AlAbdulwahab and Al-Gabbani, 2010). In addition, NMES-assisted strengthening and NMES-assisted gait were investigated (Pool et al., 2015; Özen et al., 2021). The dosage varied between 5 and 240 min per session, 3–7 days per week for 1–8 weeks.

Among the seven studies of NMES for spasticity reduction, five studies reported reduced spasticity in the antagonistic muscle when using electrical stimulation (Khalili and Hajihassanie, 2008; AlAbdulwahab and Al-Gabbani, 2010; Pool et al., 2015; Rajalaxmi et al., 2017; Özen et al., 2021). Study results included decreased resistance of the hip adductors (AlAbdulwahab and Al-Gabbani, 2010), hamstrings (Khalili and Hajihassanie, 2008; Özen et al., 2021), and gastrocnemius muscles (Pool et al., 2015; Rajalaxmi et al., 2017; Özen et al., 2021) assessed by the Modified Ashworth Scale (MAS) or Tardieu Scale; while two studies found no change in spasticity (Daichman et al., 2003; Karabay et al., 2015). Application and results of NMES-assisted spasticity reduction can be found in Tables 2, 3, 4.

### 3.4 Additional literature

Our search identified seven studies reviewing NMES as an intervention for individuals with CP, including reviews (Khamis et al., 2018; Wright M. et al., 2012), scoping reviews (Mooney and Rose, 2019; Walhain et al., 2021), and systematic reviews with meta-analysis (Salazar et al., 2019) and without meta-analysis (Chiu and Ada, 2014; Moll et al., 2017). These reviews explicitly focused on the effects of NMES on muscle morphology (Walhain et al., 2021), gait (Mooney and Rose, 2019, p. 2; Wright P. A. et al., 2012), gross motor function (Salazar et al., 2019), ankle dorsiflexion (Moll et al., 2017), activities (Chiu and Ada, 2014), and improvement in gait deviations when using FES (Khamis et al., 2018). None of the listed reviews were specific to our scoping review looking at the NMES application as a lower extremity exercise for individuals with spastic CP.

## 4 Discussion

The findings of this scoping review indicate that NMES applied to strengthening exercise, gait, and spasticity reduction demonstrate potential benefits for improving muscle physiology, neuromuscular impairments, gait patterns, and functional mobility in individuals with spastic CP. The twenty-six intervention publications, dating from 2003 to 2021, included a total of 558 individuals aged 3–57 years with CP, GMFCS levels I-IV with unilateral or bilateral involvement. The dosage of NMES intervention varied by study, as noted in Table 2. In addition, while using NMES, the exercise activities varied and included ROM, strengthening (i.e., isometric contractions, progressive resistance exercises, cycling), positioning, functional tasks, and gait. NMES included both wearable and non-wearable devices with surface electrodes, with the exception of two studies that utilized implanted electrodes (Johnston et al., 2004; Stackhouse et al., 2007).

The NMES parameters utilized in these studies included frequencies between 10 and 50 Hz, stimulation intensities between 4 and 100 mA, with typical values below 40 mA, and pulse width between 3 and 350 μs, as shown in Table 3. The most substantial variation was in pulse width, which could be attributed to individual preferences and tolerances and to the sequence of adjusting NMES parameters during treatment. Although pulse width affects muscle force production, currently, there is no evidence suggesting the range of optimal pulse width, therefore, more studies are needed. Clinical experience suggests that electrode size and adherence to the skin and pulse width contribute most to NMES comfort level.

### 4.1 Neuromuscular electrical stimulation-assisted strengthening

NMES-assisted strengthening was found to increase strength, WS, walking distance, gross motor skills, and functional mobility. Three studies reported that NMES applied during exercise provided better outcomes than exercise alone (Khalili and Hajihassanie, 2008; Arya et al., 2012; Qi et al., 2018). This may be attributed to increased sensory attention to task and motor learning. Weaker muscles are likely to gain more from NMES strengthening than stronger muscles. Physical therapy, as well as surgical preparation and recovery, provide opportunities to initiate NMES strengthening of weakened muscles. Clinical expertise suggests that voluntary contraction is an important element of strengthening and motor control versus NMES stimulation alone. The results of this scoping review found further evidence that supports the use of NMES-assisted strengthening as a clinical treatment for individuals with spastic CP. Future studies need to study the impact of NMES-assisted strengthening on biological aspects of muscle physiology and chronic health conditions in individuals with spastic CP.

Another benefit to muscle strengthening is increasing overall muscle-tendon length across the joint, which may improve ROM (Zhou et al., 2017). Increasing muscle fiber diameter through strengthening theoretically increases overall muscle-tendon length due to the diagonal muscle fiber pennation angle relative to the axis of the bone (Zhou et al., 2017). Several studies identified that muscle CSA was increased with NMES-assisted strengthening, which likely would translate to increased overall muscle-tendon length and improved ROM (Stackhouse et al., 2007; Karabay et al., 2015). Future studies need to examine the impact of NMES-assisted strengthening on overall muscle-tendon length and joint ROM.

### 4.2 Neuromuscular electrical stimulation-assisted gait

NMES-assisted gait was found to improve strength, motor control, gait pattern, and temporal-spatial parameters. Similar to NMES-assisted strengthening, the repetitive movement of walking on a treadmill combined with NMES was found to have an advantage over treadmill gait or NMES alone for improving ankle power and gross motor skills of standing and walking (Chan et al., 2004). Furthermore, another study suggested that intensive use of NMES-assisted gait in home and community settings may facilitate motor learning (Pool et al., 2014). The results of this scoping review further strengthens the evidence to support NMES-assisted gait as a clinical treatment for individuals with spastic CP. Wearable single-channel NMES units are widely available and allow for home and community use to improve foot clearance in swing; however wearable multi-channel units are not widely available. Wearable multi-channel units are needed to treat gait abnormalities other than limited foot clearance in swing. Further research and development are needed in this area.

### 4.3 Neuromuscular electrical stimulation for spasticity reduction

NMES was also found to reduce spasticity, as assessed by Tardieu or MAS in five of seven studies reviewed; one study used TENS (AlAbdulwahab and Al-Gabbani, 2010), and four studies used NMES (Khalili and Hajihassanie, 2008; Pool et al., 2015; Rajalaxmi et al., 2017; Özen et al., 2021). Corticospinal tract injury results in a loss of descending neural signal activation and inhibition. Muscle spasticity is a neuromuscular impairment that results from loss of inhibition. Further research needs to investigate the potential inhibitory effects of NMES and how to optimize spasticity reduction and duration of treatment effects. The location of ideal electrode placement along the lumbar spine, over relevant dermatomes, directly over spastic muscle, or to elicit antagonist inhibition requires further research.

### 4.4 Limitations and future research

Limitations of this scoping review include the exclusion of some NMES-related studies that did not meet inclusion criteria due to NMES treatment duration of fewer than 4 weeks, the absence of an exercise component, technology development trials for NMES-assisted gait on a treadmill (Zahradka et al., 2021) or robotics (Shideler et al., 2020). These limitations may have eliminated some evidence in the field. However, with respect to treatment duration, a recent publication recommended at least 8–20 weeks of exercise training to facilitate meaningful changes in muscle structure and improve function in individuals with CP (Moreau and Lieber, 2022). This suggests that 8–20 weeks of exercise duration may be required, and therefore, it is possible that some of the studies in our review lacked the proper dosage to produce a meaningful change. While 11 out of 26 studies in this scoping review were RCTs, further studies with larger sample sizes and more consistent protocols using CDE outcome measures are needed to move the field forward.

This scoping review indicates that further research is needed to determine optimal NMES protocols and dosage using sensitive CDE outcome measures. Furthermore, device development of wearable NMES units that can be easily applied for NMES-assisted strengthening, gait, and spasticity reduction is needed for individuals with spastic CP. Understanding the relationship between NMES strength training and functional results, as well as the optimal NMES protocol and dosage, requires research with a larger sample size and longer treatment duration (i.e., 8–20 weeks). Identifying changes in neuromuscular impairments of weakness, short-muscle tendon unit, spasticity, and impaired SMC as well as motor learning, and utilizing CDEs with careful attention to minimal clinically important differences will allow us to better comprehend the therapeutic effects of NMES. Finally, advancing new NMES technology, such as wireless multichannel NMES devices and hybrid robotic and exoskeleton NMES systems, will provide evidence-based, clinically feasible interventions for individuals with CP to improve functional mobility.

## 5 Conclusion

Findings from this scoping review provide evidence that supports the use of NMES-assisted strengthening with therapeutic exercise and cycling, NMES-assisted gait, and NMES for spasticity reduction to improve mobility in individuals with spastic CP, based on validated CDE outcome measures. Wearable and non-wearable units were utilized with surface or implanted electrodes targeting the gluteals, hip adductors, hamstrings, quadriceps, gastrocnemius, and tibialis anterior to augment exercise and mobility. NMES was found to improve muscle structure, strength, gross motor skills, gait kinematics, WS, and walking distance and reduce spasticity. Clinicians can consider NMES to be an effective treatment for individuals with spastic CP. Additional research is needed to further investigate optimal parameters, dosage, and impact of NMES on neuromuscular impairments and functional mobility in individuals with spastic CP.
